# Effects of Roasting Sweet Potato (*Ipomoea batatas* L. Lam.): Quality, Volatile Compound Composition, and Sensory Evaluation

**DOI:** 10.3390/foods10112602

**Published:** 2021-10-27

**Authors:** Yu-Jung Tsai, Li-Yun Lin, Kai-Min Yang, Yi-Chan Chiang, Min-Hung Chen, Po-Yuan Chiang

**Affiliations:** 1 Department of Food Science and Biotechnology, National Chung Hsing University, 250 Kuokuang Road, Taichung 40227, Taiwan; a0983592262@gmail.com (Y.-J.T.); chiangyichan@gmail.com (Y.-C.C.); 2 Department of Food Science and Technology, Hungkuang University, Taichung 433304, Taiwan; lylin@hk.edu.tw; 3 Department of Hospitality Management, Mingdao University, 369 Wen Hua Road, Changhua 52345, Taiwan; a9241128@gmail.com; 4 Agriculture & Food Agency Council of Agriculture Executive Yuan Marketing & Processing Division, Taipei City 10050, Taiwan; cmh@mail.afa.gov.tw

**Keywords:** sweet potato, roasting color, total acidity, sugar content, GC/MS, sensory evaluation

## Abstract

Roasting can increase the Maillard reaction and caramelization of sweet potatoes to create an attractive appearance, color, aroma, and taste, and is rapidly increasing in the commercial market. This study mainly analyzed the influence of roasting sweet potatoes, with and without the peel, on sweet potato quality and flavor characteristics combined with sensory qualities. The results showed that the a* value (1.65–8.10), browning degree (58.30–108.91), total acidity (0.14–0.21 g/100 g, DW), and maltose content (0.00–46.16 g/100 g, DW) of roasted sweet potatoes increased with roasting time. A total of 46 volatile compounds were detected and 2-furanmethanol, furfural, and maltol were identified as the main sources of the aroma of roasted sweet potatoes. A sensory evaluation based on a comprehensive nine-point acceptance test and descriptive analysis showed that roasting for 1 to 2 h resulted in the highest acceptance score (6.20–6.65), including a golden-yellow color, sweet taste, and fibrous texture. The sweet potatoes became brown after roasting for 2.5 to 3 h and gained a burnt and sour taste, which reduced the acceptance score (4.65–5.75). These results can provide a reference for increased quality in the food industry production of roasted sweet potatoes.

## 1. Introduction

Sweet potato (*Ipomoea batatas* L. Lam) is one of the most popular root crops. There are many kinds of sweet potato, all containing carbohydrates, dietary fiber, protein, minerals, and vitamins. They also contain β-carotene, chlorogenic acid, flavonoids, anthocyanins, 3,5-dicaffeoylquinic acid, and polyphenolic compounds [1]. Blessington et al. [2] noted that roasting can increase the antioxidant content, reduce sugar, and form volatile compounds to enhance the flavor. Roasting has become one of the most popular cooking treatments [3]. The high temperature and low humidity used in roasting can easily lead to protein denaturation, starch gelatinization, caramelization, and thermal degradation products with a variety of volatiles [4]. Corrales et al. [5] mentioned that a pleasant aroma is produced by the volatile compounds released by the Maillard reaction after roasting, which bears a critical influence on the overall flavor and consumer acceptance of the food.

In recent years, roasted sweet potatoes have become a snack food, moving from traditional markets to convenience store and supermarket systems, including frozen roasted sweet potato products, in many countries. This change has increased the demand for sweet potatoes. It is important to optimize the thermal process and keep the quality stable [6]. However, different processing conditions during the roasting process may greatly affect the quality of the products. Changes in appearance, aroma, flavor, taste, and aftertaste may affect consumer acceptance, and different consumer groups will be attracted by different sensory characteristics [7]. Hou et al. [8] showed a positive correlation between the color, sugar composition, free amino acids, volatile compounds, and overall acceptability of roasted sweet potatoes, especially between 2-furanmethanol and overall acceptability. Leksrisompong et al. [9] analyzed consumer preference and descriptive analysis and found that, overall, preference for roasted sweet potatoes is mainly dominated by flavor preference, followed by taste preference. In addition, studies have explored the sensory properties of sweet potato varieties with different colors, finding that yellow varieties are correlated with fibrous texture and sweet taste [10,11]. This shows that the composition of volatile compounds will affect the flavor, and thus consumer preference, and the above studies are aimed at the characteristics of different varieties of sweet potatoes before and after roasting. No study has yet investigated different roasting times or the effect of processing methods (unpeeled and peeled) on the quality of roasted sweet potatoes, so it is necessary to discuss the color, sugar composition, and volatile compounds in combination with sensory qualities of roasted sweet potatoes that have been processed in different ways.

Based on the different tastes of consumers, there is a difference between eating unpeeled and peeled sweet potatoes. If the roasting temperature is too high or the process too long, scorching, acidification, and quality deterioration result [12]. Therefore, quality standardization has become an important issue. In addition, roasting whole sweet potatoes with the peel results in better color, aroma, and flavor than other processed sweet potato products made from peeled vegetables. This study analyzed the results of different sweet potato processing methods during the roasting process. It combined the color, total acidity, sugar analysis, and volatile compound changes with sensory evaluation, and used agglomerative hierarchical clustering (AHC) and principal component analysis (PCA) to explore the consumer preference impact of quality changes in peeled and unpeeled sweet potatoes prepared with different roasting times to improve quality control and consumer competitiveness.

## 2. Materials and Methods

### 2.1. Materials Preparation

Fresh sweet potato roots (TN57) were purchased from Guarantee responsibility Qiongpu Cooperative Farm (Yunlin, Taiwan) and a similar weight of sample was selected (180 ± 20 g), washed, placed in a roaster (Model K-5, Chung Pu Baking Machinery Co., Ltd., Taichung, Taiwan) at 220 °C for (0, 0.5, 1, 1.5, 2, 2.5, 3 h), and cooled to room temperature. In this process, sweet potatoes were divided into unpeeled and peeled, which refers to baking with and without peel, respectively.

### 2.2. Color Analysis

Color values were measured by a color meter (Model NE-4000, Nippon Denshku Industries Co., Ltd., Tokyo, Japan) and were expressed in L*, a*, and b* values, where L* indicates lightness, a* represents red (>0) or green (<0), and b* expresses yellow (>0) or blue (<0). Moreover, the browning index (B.I.) was calculated by the following equation (Equations (1) and (2)) [13]:

B.I. = {100(x − 0.31)}/0.17(1)

x = (a* + 1.75L*)/(5.645L + a* − 3.012b*)(2)

### 2.3. Quality Index

#### 2.3.1. Total Starch Content

This analysis method was modified from Liu et al. [14], which was determined using a method derived from the Megazyme kit (Megazyme K-TDFR, Wicklow, Ireland).

#### 2.3.2. Sugar Composition

This analysis method was modified from Chan et al. [15]. A 1 g measure of each lyophilized powder sample was mixed with 60% ethanol. Content of sugar was analyzed by HPLC (Model L-600, Hitachi Co., Tokyo, Japan) equipped with a column (DC-613, 6 µm, 6 mm × 150 mm, Shodex Co., Tokyo, Japan). The method employed a refractive index detector (5450 RI Detector, Hitachi Co., Tokyo, Japan). The column temperature was held at 70 °C. The mobile phase was a mixture of HPLC grade acetonitrile (80%) with 1.5 mM NaOH (20%). A 10 µL measure was used for HPLC-RI and the flow rate was 1.5 mL/min [16].

#### 2.3.3. Total Acidity

This analysis was evaluated by titration with 0.1 N NaOH and expressed citric acid according to the methods by Chemists and Horwitz [17].

### 2.4. GC/MS Analysis

The volatile composition of roasted sweet potatoes was modified from Hou et al. [8] and identified with an HP-6890 gas chromatograph combined with a 5973 Turbo Pump Mass Selective Detector (MSD), and a DB-wax capillary column (60 m × 0.25 mm × 0.25 μm), were purchased by Agilent Technologies Co. (Santa Clara, CA, USA). Each 1.5 g sample was heated to 60 °C in a vial and the headspace was sampled with a DVB/CAR/PDMS fiber (Supelco Inc., Bellefonte, PA, USA) for 60 min. The injection temperature was 240 °C, the oven temperature was held at 40 °C for 1 min, and was increased to 160 °C at a rate of 2 °C/min and further increased to 240 °C at a rate of 5 °C/min. Retention indices were calculated from the retention times of *n*-alkanes (C_8_–C_25_) that were run under the same chromatographic conditions. The identification of volatile compounds was compared with the mass spectral data obtained with those in the NIST library.

### 2.5. Sensory Evaluation

Sensory evaluation was measured by the method of previous studies [8,15,18]. In this study, 60 panelists (students and staff of NCHU) were inducted in the evaluation room in batches. Most of these panelists were between 19 and 25 years old, including 14 males (23%) and 46 females (67%). A total of 25 members (42%) were accustomed to eating roasted sweet potatoes with their peels, and 35 (58%) were not. Samples were evaluated on a 9-point hedonic scale ranging from 1 (extremely dislike) to 9 (extremely like) [19]. The results of overall acceptability were based on the comprehensive sensory attributes, including visual, aroma, flavor, texture, and aftertaste. In addition, the comprehensive sensory attributes definition of the description analysis test needed to be mentioned three times and each time for 30 min to ensure the correct understanding of the sensory attributes’ description terminology. This evaluation took 30–60 min and these participants needed to clean their palates with crackers and water before tasting the next new sample (Table A1) [11,19,20].

### 2.6. Statistical Analysis

Each test was carried out in triplicate and the data are expressed as mean ± standard deviation. One-way analysis of variance (ANOVA) was conducted using Duncan’s multiple range test and correlation analysis was performed using SPSS software (version 19 (2018), IBM Co., Armonk, NY, USA). The data were subjected to an AHC with squared Euclidean distances. Subsequently, the data were analyzed using PCA combined with VARIMAX rotation. For the AHC and PCA analyses, XLSTAT software (version in 2020, Addinsoft Institute Inc., New York, NY, USA) was used.

## 3. Results and Discussion

### 3.1. Appearance and Color Analysis

**Figure 1:**
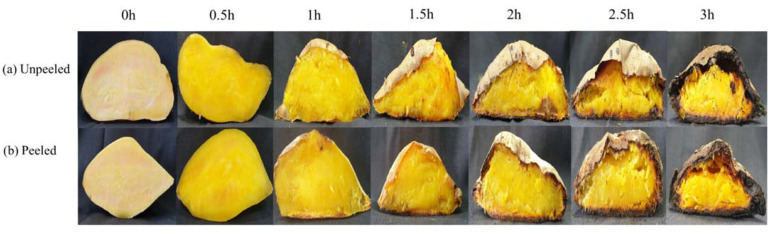
The appearance of roasted sweet potatoes. (**a**) Unpeeled; (**b**) Peeled.

Sweet potatoes are mainly composed of starch, crude protein, crude fiber, and polysaccharides. They are easily heated during the roasting process to gelatinize the starch, caramelize, and undergo the Maillard reaction to create a good color and flavor [20]. The cut surface of fresh sweet potato has a milky white, firm appearance and structure. With the extension of roasting time, the internal temperature rises from 27 °C to 101 °C, causing the sweet potato to “grill”, forming a softer texture and gradually more golden color. The color change to yellow is relatively stable after roasting for 1 to 1.5 h, but after roasting for 2 to 2.5 h, the outer peel of the sweet potato is likely to be scorched, and the flesh will shrink and change due to the evaporation of water. A peeled sweet potato will form a crusty surface due to the evaporation of water after roasting for 1 h. The cooking phenomena were more extreme after roasting for 3 h (Figure 1). Fresh sweet potatoes have higher brightness due to higher water and starch content. The L*, a*, b*, and B.I. values of unpeeled sweet potato were 70.35, 4.40, 28.99, and 56.38, and those of peeled sweet potato were 67.50, 5.51, 30.84, and 65.30. As the roasting time increased (0.5 to 3.0 h), the L* value of peeled sweet potato decreased from 49.90 to 40.98, and that of peeled sweet potato from 47.33 to 44.62; the a* value increased significantly after 1.5 h of roasting, and the b* value showed a significant increase after 1.5 h of roasting (Table 1). During roasting, peeled sweet potatoes’ starch and polysaccharides will form a secondary crusty peel. The sweet potatoes undergoing the two treatments were dark brown due to the decrease in L* value and the increase in a* value, and the degree of browning rises rapidly [8]. After roasting for more than 2.5 h, the two sweet potatoes exhibited similar roasting effects, and both were brown with a hard-shell surface. There was no significant difference (*p* > 0.05) in B.I. and L * values, which is mainly due to roasting at high temperatures for a long time. Roasting causes caramelization and the Maillard reaction, producing dark polymers [8,21] and affecting the acceptability of the final products [22,23].

### 3.2. Quality Index

Changes occurred in sugar composition during sweet potato roasting. The total starch content before roasting was around 64.62 g/100 g (Table 2). As the roasting time increased, starch gradually transformed into monosaccharides and disaccharides, so the total starch content decreased significantly (64.62 g–51.10 g/100 g). However, there was no significant difference in the total starch content of sweet potatoes after ripening. This may be due to the use of heat-stable amylase to convert starch into maltodextrin during the determination of total starch content, and then the use of high-purity amyloglucosidase to quantify glucose, which is degraded by dextrin [24]. While heat treatment changes the structure of starch, the structure of glucose does not change [25]. Chan et al. [15] pointed out that sweetness is one of the most important factors in determining the overall appeal of roasted sweet potatoes. In unroasted sweet potatoes, sucrose content is the highest (11.49 g/100 g, DW), followed by glucose (2.23 g/100 g, DW), and fructose (1.60 g/100 g, DW), as Lai et al. [26] reported, while the maltose content is affected by heat treatment and β-amylase, which decomposes starch into maltose and wheat maltodextrin, so the maltose content tends to increase rapidly after roasting [27]. Among the tested samples, the highest maltose content (51.82 g–55.28 g/100 g, DW) was found in sweet potatoes that were peeled and roasted for 1 to 2 h, which may be the cause of the difference in the volatile compound content found in sweet potatoes under the subsequent high temperature and long-term roasting treatment. However, the content of sucrose decreased (12.51 g–10.01 g/100 g, DW) after roasting for 1.5 h. Sucrose may participate in caramelization during heat treatment to produce caramel and polymerized dark substances, which will affect the sweet potato color, aroma, and flavor [8].

Roasted sweet potatoes tended to have a sour taste when over-roasted (Table 2). The group that was roasted for 2.5 to 3 h had the highest total acidity value (0.18–0.21 g/100 g, DW) and the total acidity of peeled potatoes was significantly higher than unpeeled samples (0.21 g/100 g and 0.19 g/100 g, DW, respectively), which may be related to water loss, sugar molecule cleavage, and interactions between polyphenol compounds and polysaccharides in the cell wall [12] Other studies have reported that the pH and total acidity of sweet potatoes will also change during the roasting process [28,29,30].

### 3.3. GC/MS Analysis

**Figure 2:**
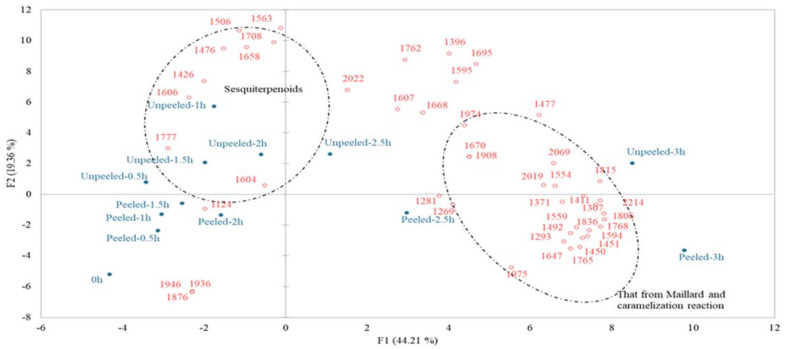
PCA plots of the volatile compound formation of sweet potato during roasting process; the hollow dots indicate the compounds responsible for the perceived aroma of sweet potatoes.

The aroma and flavor of roasted sweet potatoes mainly come from thermal pyrolyzes, such as the thermal release of terpene glycosidic bonds, carotenoid degradation, caramelization, the Maillard reaction, and Strecker degradation [4,31]. A total of 46 volatile compounds were detected in roasted sweet potatoes (Table 3), including sesquiterpenoids and their oxides (13), furan compounds (12), ketones (6), nitrogen-containing compounds (4), and other volatile compounds (11), including benzeneacetaldehyde, acetic acid, and γ-decalactone, which together form the unique flavor of roasted sweet potatoes. The aroma compounds detected in fresh sweet potatoes mainly come from nerolidol (floral odor), trans-β-ionone (violet-like, floral odor), and γ-decalactone (fruity odor) [32,33]. After roasting for 0.5 to 2 h, the aroma is mainly derived from trans-β-ionone, β-damascenone (sweet odor) [34,35], which is formed by the degradation of carotenoids, and benzeneacetaldehyde (floral, honey-like), which is formed by the degradation of phenylalanine in Strecker degradation [36]. Carbohydrates degrade into dark polymers and form 5-hydroxymethylfurfural (sweet odor), furfural (roasted nut odor), 2-furanmethanol (caramel-like, roasted odor), and maltol (caramel-like odor). These and other furans and their derivatives are important aroma compounds in roasted sweet potatoes and often appear with the Maillard reaction, which makes the aroma composition produced by the Maillard reaction more complex [37,38]. 2-furanmethanol, a source of sweetness and caramel odor, is positively correlated with overall preference [8]. However, after a long roasting time (2.5 to 3 h), the α-dicarbonyl compound and amino acids undergo Strecker degradation and participate in the Maillard reaction after polymerization, which affects the color and aroma by forming 2-pyrrolecarbaldehyde, 4-methyl-5-thiazoleethanol, and 1-(1*H*-pyrrol-2-yl)-ethanone, among other nitrogen-containing compounds providing roasting aromas [39]. The roasting process may degrade monosaccharides or oxidize aldehydes during the caramelization process; acetic acid generates a sour taste, which in turn changes the quality of roasted sweet potatoes and affects consumer preference [40]. Qin et al. [41] pointed out that aroma plays an important role in the overall flavor and sensory acceptance to consumers. To explain the aroma characteristics more completely, many studies now use PCA to analyze GC/MS data and identify important volatile compounds. By reducing the multi-dimensional data, it is easy to observe the differences between samples [42]. In this experiment, PCA was used to determine the important volatile compounds of peeled and unpeeled sweet potatoes roasted for different times to establish the relationship between the treatment method and the volatile compounds [43]. The first. principal component (F1 of two dimensions) and the second principal component (F2) combined can explain about 63.56% of the raw data (Figure 2). F1 accounts for 44.21%, explaining the distribution of the roasting time. The group roasted for 2 h is distributed in the negative direction of the *X*-axis, and the group roasted for 2.5 to 3 h is distributed in the positive direction of the *X*-axis, and displays increasing pyrolysis products such as β-damascenone and furan derivatives (Table 3, No. 5, 8, 14, 16, 17, 27, 31, 36, 38, 41, 42, 46) and N-containing compounds (Table 3, No. 29, 32, 34, 45). F2 accounted for 19.36%, explaining the difference between the two processing conditions. In Figure 2, the unpeeled sweet potatoes are distributed in the positive direction of the *Y*-axis, and they are rich in sesquiterpene compounds and carotenoid oxidation products. The peeled sweet potatoes are distributed in the negative direction of the *Y*-axis. In addition, during the roasting process, AHC was used to identify the aroma characteristics of three clusters. Cluster I contained unroasted and peeled potatoes roasted for 0.5 to 2 h; cluster II contained unpeeled potatoes roasted for 0.5 to 2.5 h and peeled potatoes roasted for 2.5 h; cluster III contained unpeeled and peeled potatoes roasted for 3 h, which are positively correlated with caramelization and the formation of Maillard reaction products. In addition, the distribution of volatile compounds shows that as the roasting time increased, the thermal degradation cracked and polymerized more organic compounds to generate more volatile compounds, thus increasing the diversity of volatile compounds [44,45].

### 3.4. Sensory Evaluation

**Figure 3:**
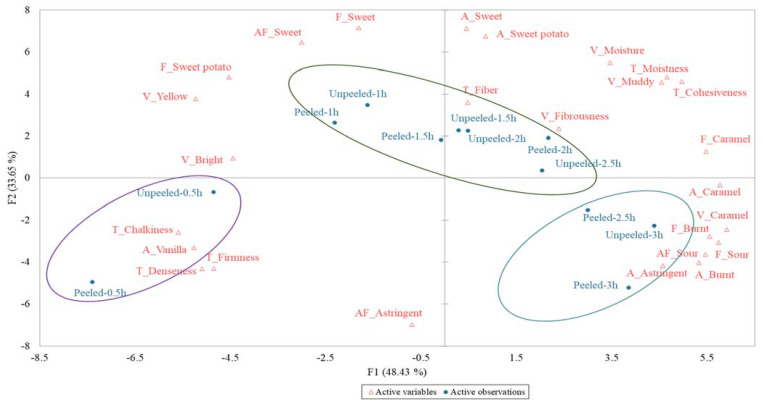
Principle component analysis with (F1 = 48.43% and F2 = 33.65%) variability of all descriptive terms explained for all samples. Abbreviations before attributes: V, visual; A, aroma; F, flavor; T, texture; AF, aftertaste.

To clarify the impact of quality changes on consumer preferences, acceptance, and descriptive analyses were conducted. Roasting for 1 to 2 h resulted in the highest overall preference score (6.25–6.65), followed by roasting for 2.5 h (5.71, 5.75), 3 h (4.65, 5.53), and 0.5 h of roasting resulted in the lowest overall preference score (4.96, 3.84). The acceptance results of different roasting times for unpeeled and peeled potatoes trended similarly (Table 4). The largest difference of overall preference score for peeled and unpeeled sweet potatoes in aroma, flavor, taste, and aftertaste was found after roasting for 0.5 h (4.96 and 3.84, respectively). As the roasting time increased, the overall preference score increased. However, when roasting for 1 h, the aroma of unpeeled roasted potatoes scored significantly higher than that of peeled roasted potatoes (6.51 and 5.75, respectively), which may be related to the higher content of benzeneacetaldehyde created, and the greater content of β-ionone and some sesquiterpenes in roasting unpeeled potatoes contributed sweetness and floral fragrance. After further roasting (2.5 to 3 h), the degradation of monosaccharides caused a reduction in sweetness, an increase in total acidity, and the formation of acetic acid, which significantly reduced the overall preference score. The total acidity of peeled roasted sweet potatoes was higher than unpeeled (0.21 g/100 g and 0.19 g/100 g, DW, respectively), so their overall acceptance score (4.65 and 5.53), flavor score (4.15 and 5.45), and mouthfeel score (5.44 and 6.18) were significantly lower. The overall preference score of the roasted sweet potatoes is affected by the flavor and mouthfeel scores [9]. Descriptive analysis was performed according to Pareto’s 80/20 rule, which has been used in many sensory studies [46]. The sensory characteristics of roasted sweet potatoes were further explored by six appearance attributes, six aroma attributes, five flavor attributes, six taste attributes, and three aftertaste attributes (Figure 3). The results showed that 12 samples could be distinguished by PCA as three clusters, the F1 explains about 48.43% of the data, and the F2 explains about 33.65% of the data. Cluster I was the group of samples roasted for 0.5 h. Due to the short roasting time, this cluster had an obvious vanilla aroma, denseness, firmness, chalkiness, and astringent aftertaste. The above-mentioned negative sensory characteristics made it have the lowest overall preference score (aroma, flavor, texture, and aftertaste). Cluster II included unpeeled samples roasted for 1 to 2.5 h and peeled samples roasted for 1 to 2 h. As the group in this cluster had higher yellowness (b* value), the maltose, furfural, 2-furanmethanol, and maltol combined with sesquiterpenoids to form the unique aroma of sweet potato, which enabled consumers to notice the obvious yellow color, fibrous texture, moisture, and sweet potato flavor, sweetness, caramel flavor, and sweet aftertaste in this cluster. Roasted sweet potato with these sensory characteristics can increase consumer preference, which is similar to the sensory attribute results of the yellow-flesh sweet potato mentioned in [10,11]. Cluster III contained unpeeled samples roasted for 3 h and peeled samples roasted for 2.5 to 3 h. This cluster was most affected by caramelization and the Maillard reaction, which causes carbohydrates to crack and polymerize to form caramel and melanin [47,48], increasing the browning index (105.54–108.91), total acidity (0.18 g–0.21 g/100 g, DW), and furans content (Table 3, No. 5, 8, 14, 16, 17, 27, 31, 36, 38, 41, 42, 46). Although furans can exhibit malt and sweet roasting aroma, the significant increase at higher roasting levels produces a burnt aroma [49]. In addition, N-containing compounds (Table 3, No. 29, 32, 34, 45) and acetic acid (Table 3, No. 4) produced a readily apparent burnt smell and sourness, causing quality deterioration and a significant reduction in consumers’ overall preference for samples in this cluster, as they noticed the obvious changes of caramel color, caramel aroma, burnt aroma, sour aroma, and burnt flavor. Besides, after roasting 1–2 h at 220 °C, the results of the overall acceptability were around 6.25–6.65 (point 6 indicates like slightly), which corresponds to cluster II in Figure 3. In consumer sensory evaluation, the overall score of cluster II was higher than other clusters (I and III). With increased time of roasting process from 2 h to 3 h, descriptive attributes changed from sweet, sweet potato, and fiber to caramel, sour, and fibrousness. Lower overall acceptability (4.65–5.75) corresponds that over-caramelization and starch liquefying of sweet potatoes [8,50].

## 4. Conclusions

This study explored the effect of processing methods on the quality of roasted sweet potatoes as measured by consumer preference. This study found that the ideal roasting time has the following effects: starch hydrolysis and thermal cracking reactions (carotenoid degradation, caramelization, the Maillard reaction, and Strecker degradation) change the flesh color from milky white to golden yellow, then to brown, and the browning index had an increasing trend with the roasting time and significant increases in maltose, β-ionone, benzeneacetaldehyde, furfural, maltol, 2-furanmethanol, and sesquiterpenoids, providing fruity, sweet, and caramel-like odors and adding a unique flavor to the roasted sweet potatoes. These results also show that the difference in the composition of volatile compounds due to the degradation of various organic compounds is mainly determined by the roasting time, and the roasting time also has a more significant influence on the overall preference of consumers than whether the sweet potatoes are peeled or not. Comprehensive production cost considerations, quality analysis, and sensory evaluation tests show that the best roasted sweet potato quality is obtained by roasting for 1 to 2 h.

## Figures and Tables

**Table 1:** The influence of different roasting treatments on the color change of sweet potatoes.

Time	L*		a*		b*		B.I.	
(h)	Unpeeled	Peeled	Unpeeled	Peeled	Unpeeled	Peeled	Unpeeled	Peeled
0	70.35 ± 0.27 ^gA^	67.50 ± 0.33 ^fA^	4.40 ± 0.16 ^cA^	5.51 ± 0.08 ^dA^	28.99 ±0.12 ^eA^	30.84 ± 0.41 ^eA^	56.38 ± 0.62 ^aA^	65.30 ± 1.06 ^aA^
0.5	49.90 ± 0.16 ^eB^	47.33 ± 0.23 ^cA^	1.65 ± 0.07 ^aA^	3.33 ± 0.35 ^cB^	27.94 ± 0.15 ^dB^	26.90 ± 0.41 ^bA^	80.39 ± 0.29 ^bA^	85.08 ± 1.80 ^bB^
1	50.83 ± 0.38 ^fB^	49.85 ± 0.83 ^eA^	1.47 ± 0.10 ^aB^	0.54 ± 0.26 ^aA^	29.83 ± 0.19 ^eA^	29.91 ± 0.28 ^fA^	85.55 ± 0.60 ^cA^	86.77 ± 1.43 ^bA^
1.5	45.16 ± 0.59 ^dA^	48.73 ± 0.67 ^dB^	3.27 ± 0.62 ^bB^	2.22 ± 0.30 ^bA^	26.58 ± 0.39 ^cA^	30.45 ± 0.33 ^gB^	89.43 ± 1.63 ^dA^	95.01 ± 1.96 ^cB^
2	39.62 ± 0.37 ^aA^	41.14 ± 0.22 ^aB^	5.98 ± 0.29 ^dA^	6.10 ± 0.51 ^eB^	23.54 ± 0.20 ^aA^	25.81 ± 0.18 ^aB^	96.92 ± 0.37 ^eA^	103.69 ± 2.03 ^dB^
2.5	42.23 ± 0.61 ^cA^	42.76 ± 0.44 ^bA^	7.63 ± 0.11 ^eB^	7.06 ± 0.32 ^fA^	25.25 ± 0.65 ^bA^	26.26 ± 0.32 ^bB^	104.17 ± 2.73 ^fA^	108.07 ± 0.64 ^dA^
3	40.98 ± 3.03 ^bA^	44.62 ± 1.08 ^aA^	8.10 ± 0.15 ^fB^	7.52 ± 0.23 ^gA^	22.08 ± 0.87 ^cA^	27.60 ± 0.46 ^dB^	106.32 ± 4.74 ^fA^	103.34 ± 1.25 ^eA^

1. Each value is expressed as mean ± standard deviation (*n* = 3). 2. Values (a–g) with different letters within the same column (*p* < 0.05). 3. Values (A,B) with different letters within the same roasting time (*p* < 0.05).

**Table 2:** Analysis of sugar composition and total titratable acidity of sweet potatoes by different roasting treatments.

Time	Total Starch		Fructose		Glucose		Sucrose		Maltose		Total Titratable Acidity	
(h)	(g/100 g)		(g/100 g, DW)		(g/100 g, DW)		(g/100 g, DW)		(g/100 g, DW)		(g/100 g, DW)	
Type	Unpeeled	Peeled	Unpeeled	Peeled	Unpeeled	Peeled	Unpeeled	Peeled	Unpeeled	Peeled	Unpeeled	Peeled
0	64.62 ± 0.46 ^d^	64.62 ± 0.46 ^d^	1.60 ± 0.29 ^a^	1.82 ± 0.24 ^ab^	2.23 ± 0.10 ^a^	2.60 ± 0.18 ^a^	11.49 ± 0.02 ^c^	12.68 ± 1.10 ^a^	ND	ND	0.14 ± 0.01 ^b^	0.14 ± 0.01 ^abc^
0.5	50.57 ± 0.13 ^a^	51.84 ± 0.06 ^a^	1.70 ± 0.06 ^a^	1.56 ± 0.12 ^a^	2.54 ± 0.02 ^ab^	2.62 ± 0.05 ^ab^	10.63 ± 0.21 ^ab^	11.12 ± 0.46 ^a^	41.45 ± 0.45 ^a^	45.92 ± 2.21 ^a^	0.11 ± 0.01 ^a^	0.09 ± 0.01 ^a^
1	53.36 ± 0.06 ^c^	53.94 ± 0.57 ^c^	1.87 ± 0.06 ^ab^ *	1.97 ± 0.26 ^ab^ *	2.61 ± 0.00 ^ab^	3.19 ± 0.43 ^cd^	11.66 ± 0.34 ^bc^	11.79 ± 0.69 ^a^	39.04 ± 0.16 ^a^ *	52.70 ± 2.69 ^ab^ *	0.11 ± 0.01 ^a^	0.11 ± 0.01 ^ab^
1.5	52.28 ± 0.03 ^b^	53.38 ± 0.42 ^bc^	2.39 ± 0.01 ^c^ *	2.13 ± 0.26 ^b^ *	3.60 ± 0.23 ^c^	3.37 ± 0.02 ^c^	12.01 ± 0.14 ^bc^	12.51 ± 0.91 ^a^	39.79 ± 0.37 ^a^ *	55.28 ± 3.30 ^b^ *	0.13 ± 0.01 ^b^	0.14 ± 0.01 ^abc^
2	51.79 ± 0.06 ^b^	52.47 ± 0.25 ^abc^	1.94 ± 0.02 ^b^	2.00 ± 0.47 ^b^	2.79 ± 0.23 ^ab^	3.38 ± 0.28 ^c^	11.63 ± 0.06 ^bc^	12.09 ± 1.40 ^a^	39.71 ± 0.93 ^a^ *	51.82 ± 2.70 ^ab^ *	0.14 ± 0.01 ^b^	0.16 ± 0.01 ^abc^
2.5	51.20 ± 0.90 ^b^	52.90 ± 0.93 ^abc^	2.02 ± 0.09 ^b^	1.98 ± 0.15 ^ab^	2.75 ± 0.01 ^ab^	3.01 ± 0.14 ^bcd^	11.12 ± 0.24 ^abc^	12.36 ± 2.09 ^a^	40.00 ± 0.71 ^a^ *	49.82 ± 1.69 ^ab^ *	0.14 ± 0.01 ^b^ *	0.18 ± 0.01 ^bc^ *
3	51.10 ± 0.91 ^b^	51.42 ± 0.73 ^ab^	2.05 ± 0.05 ^b^ *	1.77 ± 0.13 ^ab^ *	2.89 ± 0.12 ^b^	2.72 ± 0.19 ^abc^	10.01 ± 0.01 ^a^ *	10.79 ± 0.04 ^a^ *	38.13 ± 0.20 ^a^	46.16 ± 3.00 ^a^	0.19 ± 0.01 ^c^ *	0.21 ± 0.01 ^c^ *

1. Each value is expressed as mean ± standard deviation (*n* = 3). 2. Values (a–d) with different letters within the same column. (*p* < 0.05). 3. ND: not detected. (*) Indicates the significant difference in the same roasting time. 4. DW: dry weight.

**Table 3:** Volatile compounds of sweet potatoes identified during roasting process.

No	Compound ^a^	RI ^b^	No	Compound ^a^	RI ^b^
1	Tetradecane	1124	24	Corylone	1647
2	Pentadecane	1269	25	*cis*-muurola-3,5-diene	1658
3	Nonanal	1281	26	β-Damascenone	1668
4	Acetic acid	1293	27	Furaneol	1670
5	Furfural	1307	28	*trans*-Calamenene	1695
6	3-Methyl-tridecane	1371	29	*N*-Methylsuccinimide	1708
7	Copaene	1396	30	Butylated Hydroxytoluene	1762
8	5-Methyl-2-furaldehyde	1411	31	Maltol	1765
9	Cyperene	1426	32	1-(1*H*-pyrrole-2-yl)-ethanone	1768
10	γ-Butyrolactone	1450	33	*trans*-ß-Ionone	1777
11	4-Hydroxybutyric acid	1451	34	2-Pyrrolecarbaldehyde	1806
12	Benzeneacetaldehyde	1476	35	Pantolactone	1815
13	Pristane	1477	36	5-Methyl tetrahydrofurfuryl alcohol	1836
14	2-Furanmethanol	1492	37	Nerolidol	1876
15	α-Himachalene	1506	38	3,5-dimethyl-2,4(3*H*,5*H*)-Furandione	1908
16	5-methyl-2-furanmethanol	1554	39	8α-*H*-Secoeudesmanolide	1936
17	2(5H)-Furanone	1559	40	γ-Decalactone	1946
18	γ-Gurjunene	1563	41	Rosefuran	1974
19	α-Ionol	1594	42	5-Acetoxymethyl-2-furaldehyde	1975
20	α-Guaiene	1595	43	2,3-Dihydro-3,5-dihydroxy-6-methyl-4h-pyran-4-one	2019
21	α-Muurolene	1604	44	Butyl 2 heptenate	2022
22	α-Humulene	1606	45	4-Methyl-5-thiazolethanol	2069
23	α-Bisabolol	1607	46	5-Hydroxymethylfurfural	2214

^a^ Identified via comparison of the mass spectra with the RI. ^b^ RI: Retention index.

**Table 4:** The average value of consumer acceptance of the sensory quality of sweet potato with different roasting treatments.

Sample	Overall	Visual	Aroma	Flavor	Texture	Aftertaste
Unpeeled-0.5 h	4.96 ± 1.26 ^c^ ***	5.65 ± 1.38 ^a^	5.35 ± 1.08 ^b^ ***	4.95 ± 1.30 ^d^ ***	4.98 ± 1.56 ^b^ ***	5.20 ± 1.15 ^cd^ ***
Unpeeled-1 h	6.55 ± 1.37 ^a^	6.24 ± 1.23 ^a^	6.51 ± 1.40 ^a^ **	6.49 ± 1.43 ^ab^	6.47 ± 1.33 ^a^	6.71 ± 1.18 ^a^
Unpeeled-1.5 h	6.20 ± 1.39 ^ab^	5.71 ± 1.47 ^a^ *	6.27 ± 1.35 ^a^	6.31 ± 1.49 ^abc^	5.87 ± 1.44 ^a^ *	5.91 ± 1.55 ^abc^
Unpeeled-2 h	6.25 ± 1.46 ^ab^	6.25 ± 1.39 ^a^	6.67 ± 1.09 ^a^	6.56 ± 1.46 ^a^	6.47 ± 1.55 ^a^	6.25 ± 1.54 ^ab^
Unpeeled-2.5 h	5.71 ± 1.61 ^bc^	5.87 ± 1.50 ^a^	6.56 ± 1.45 ^a^	5.65 ± 1.95 ^bcd^	6.02 ± 1.67 ^a^	5.49 ± 1.59 ^bcd^
Unpeeled-3 h	5.53 ± 1.89 ^bc^ *	5.67 ± 1.60 ^a^	6.16 ± 1.55 ^a^	5.45 ± 2.09 ^cd^ **	6.18 ± 1.66 ^a^ *	4.95 ± 1.95 ^d^
Peeled-0.5 h	3.84 ± 1.32 ^d^ ***	5.18 ± 1.25 ^c^	4.47 ± 1.12 ^c^ ***	3.85 ± 1.38 ^c^ ***	3.38 ± 1.34 ^c^ ***	4.18 ± 1.40 ^c^ ***
Peeled-1 h	6.35 ± 1.27 ^ab^	5.91 ± 1.25 ^abc^	5.75 ± 1.00 ^b^ **	6.45 ± 1.26 ^a^	6.31 ± 1.36 ^a^	6.25 ± 1.28 ^a^
Peeled-1.5 h	6.60 ± 1.15 ^a^	6.49 ± 1.44 ^a^ *	6.20 ± 1.46 ^ab^	6.47 ± 1.26 ^a^	6.53 ± 1.29 ^a^ *	6.13 ± 1.32 ^ab^ *
Peeled-2 h	6.65 ± 1.25 ^a^	6.51 ± 1.18 ^a^	6.87 ± 1.26 ^a^	6.67 ± 1.52 ^a^	6.53 ± 1.39 ^a^	6.31 ± 1.30 ^a^
Peeled-2.5 h	5.75 ± 1.65 ^b^	6.02 ± 1.35 ^ab^	6.51 ± 1.26 ^a^	5.45 ± 1.95 ^b^	6.24 ± 1.40 ^a^	5.31 ± 1.90 ^b^
Peeled-3 h	4.65 ± 1.91 ^c^ *	5.58 ± 1.66 ^bc^	5.69 ± 1.94 ^b^	4.15 ± 2.01 ^c^ **	5.44 ± 1.77 ^b^ *	4.24 ± 1.90 ^c^ *

1. Each value is expressed as mean ± standard deviation (*n* = 60). 2. Values (a–d) with different letters within the same column (*p* < 0.05). (*) Indicates significant difference in the same roasting time. (* *p* < 0.05, ** *p* < 0.01, *** *p* < 0.0001).

## Data Availability

The datasets generated for this study are available on request to the corresponding author.

## Appendix A

**Table A1:** Sensory attributes of sweet potatoes.

Attribute		Description
Visual	Yellow	Flesh that is yellow in colour.
	Caramel	Appearance associated with brown sugar.
	Fibrousness	Amount of stringy fibers present.
	Moisture	Appearance that is moist.
	Muddy	Appearance that is muddy.
	Bright	Appearance that is bright.
Aroma	Sweet	Aromatic like sugar.
	Caramel	Aromatic associated with brown sugar.
	Sour	Aromatic associated with acid.
	Burnt	An aromatic associated with vegetables that were burnt while cooking.
	Sweet potato	Aromatic associated with cooked sweet potato of TNG57.
	Vanilla	Aromatic notes associated with damp soil, wet foliage or slightly undercooked potatoes. or In-mouth aromatic associated with vanilla and vanillin.
Flavor	Sweet potato	Flavor notes associated with the taste of cooked TNG57.
	Sweet	Tastes like sugar.
	Caramel	Flavor associated with brown sugar.
	Sour	Basic taste stimulated by acid.
	Burnt	The degree of browning or brown spots due to roasting.
Texture	Moistness	The amount of moistness/wetness of the sample in the mouth.
	Cohesiveness	Degree to which sample holds together after chewing.
	Denseness	The solidness/compactness of the sample.
	Firmness	Degree to which the sample retains its shape after lightly squeezing it.
	Chalkiness	Degree to which the mouth feels chalky, like raw potato, very fine particles, often perceived on the roof of the mouth.
	Fiber	The quality of being fibrous.
Aftertaste	Sweet	An aftertaste that leaves a sweetness on the tongue and in the mouth that is pleasant.
	Sour	Aftertaste associated with brown sugar.
	Astringent	Sensation of drying, drawing and/or puckering of any of the mouth surfaces.
